# Supplementary material for: Rapid Selective Detection and Quantification of β-Blockers Used in Doping Based on Molecularly Imprinted Nanoparticles (NanoMIPs)
Source: Polymers (Basel). 2022 Dec 11;14(24):5420. doi: 10.3390/polym14245420 (PMC9787605; doi:10.3390/polym14245420)
Supplement: Supplementary file 1 [file polymers-14-05420-s001.zip › polymers-1960056-supplementary.pdf]

Suplementary Information

Atenolol

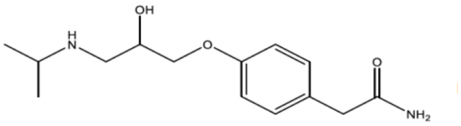

Labetalol

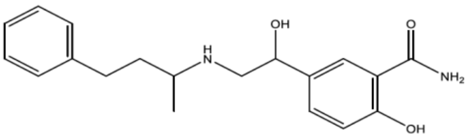

S1. Chemical structures of atenolol and labetalol. At the top is atenolol with molecular formula $C_{14}H_{22}N_2O_3$, in the lower part is labetalol with molecular formula $C_{19}H_{24}N_2O_3$.

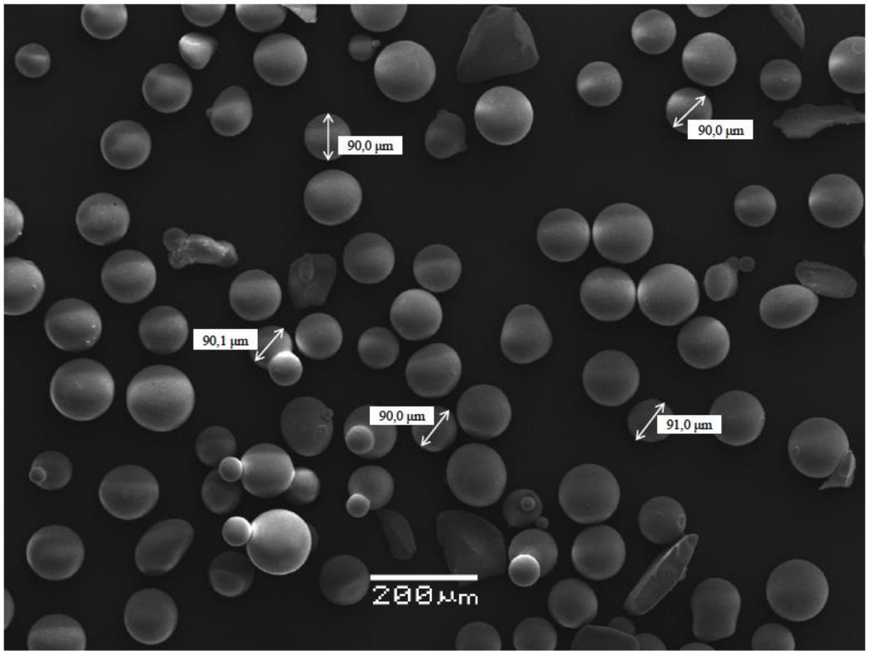

S2. SEM of the GBs used for the solid-phase synthesis of nanoMIPs.

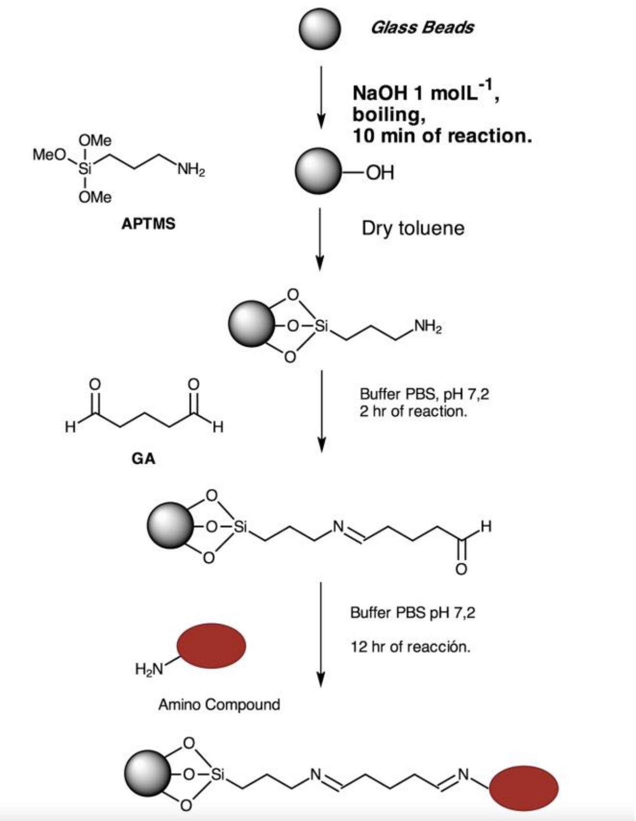

S3. Surface activation of glass beads and immobilization process of the target molecules (atenolol and labetalol) through a imine coupling chemistry.

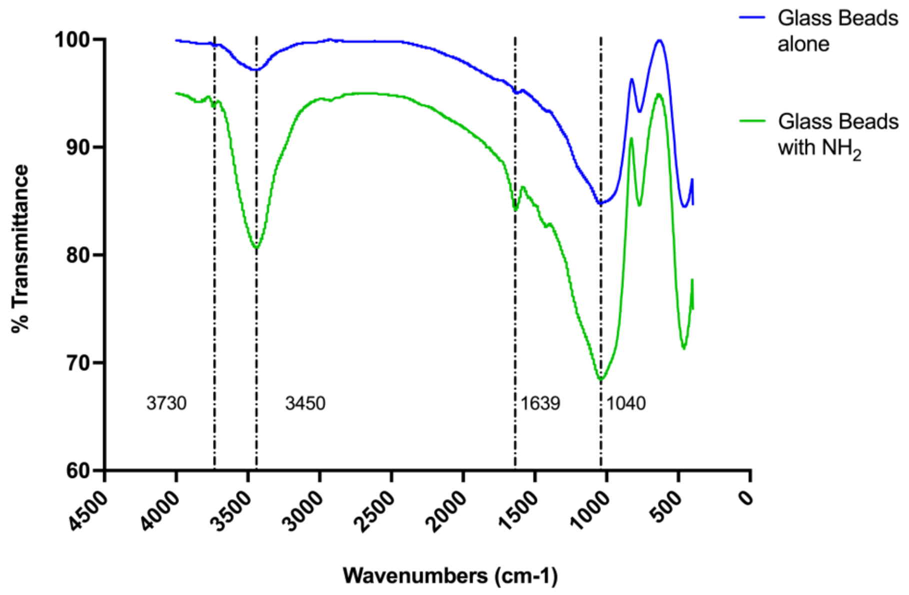

S4. FT-IR spectra of unmodified GBs and amine-functionalized GBs. Spectrum marked in blue indicates standard GBs (GBs-bare) and green color corresponds to the spectrum of GBs after reaction with APTMS (GBs-NH_2).

The figure presents the FT-IR spectrum of standard GBs (SiO_2), marked in blue color (GBs-bare), and the spectrum of GBs modified with APTMS (SiO_2-APTMS), marked in green color (GBs-NH_2). They were used to identify certain specific functional groups in a molecular structures. The absorption bands represent areas of the spectrum where occur certain bond vibrations. According to scientific data, the absorption band in the region around 1040 cm^{-1} corresponds to the vibration of $(SiO)_n$ groups, the vibration band at 3440 cm^{-1} is attributed to the presence of hydroxyl groups (-OH) and at and 1633 cm^{-1} surface silanol groups with hydrogen bond and the remaining adsorbed water molecules . The band at 973 cm^{-1} is assigned to Si-OH stretching. The bands at 787 cm^{-1} and 467 cm^{-1} are assigned to Si-O-Si stretching and Si-O-Si bending. New bands can be found after the modification with the coupling agent APTMS. The presence of NH_2 groups is reflected by the vibration band at 1421 cm^{-1}, 1421 cm^{-1} and 3770 cm^{-1}. The results show that APTMS has been successfully immobilized and the surface of GBs contained primary amine groups.

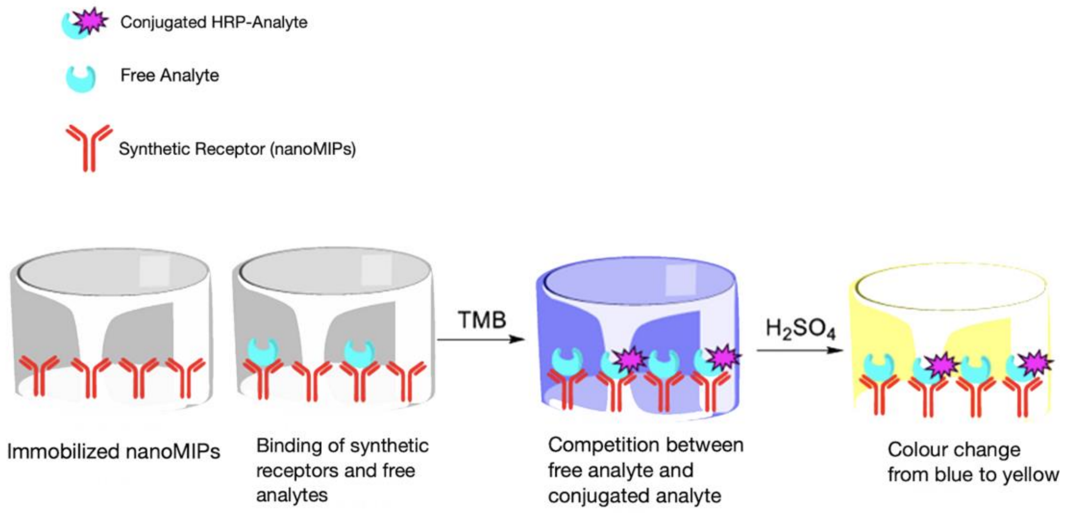

S5. Schematic illustration of a competitive pseudo-ELISA including nanoMIPs immobilized on the microplate surface. The figure presents binding competition between free analyte and analyte conjugated with HRP.

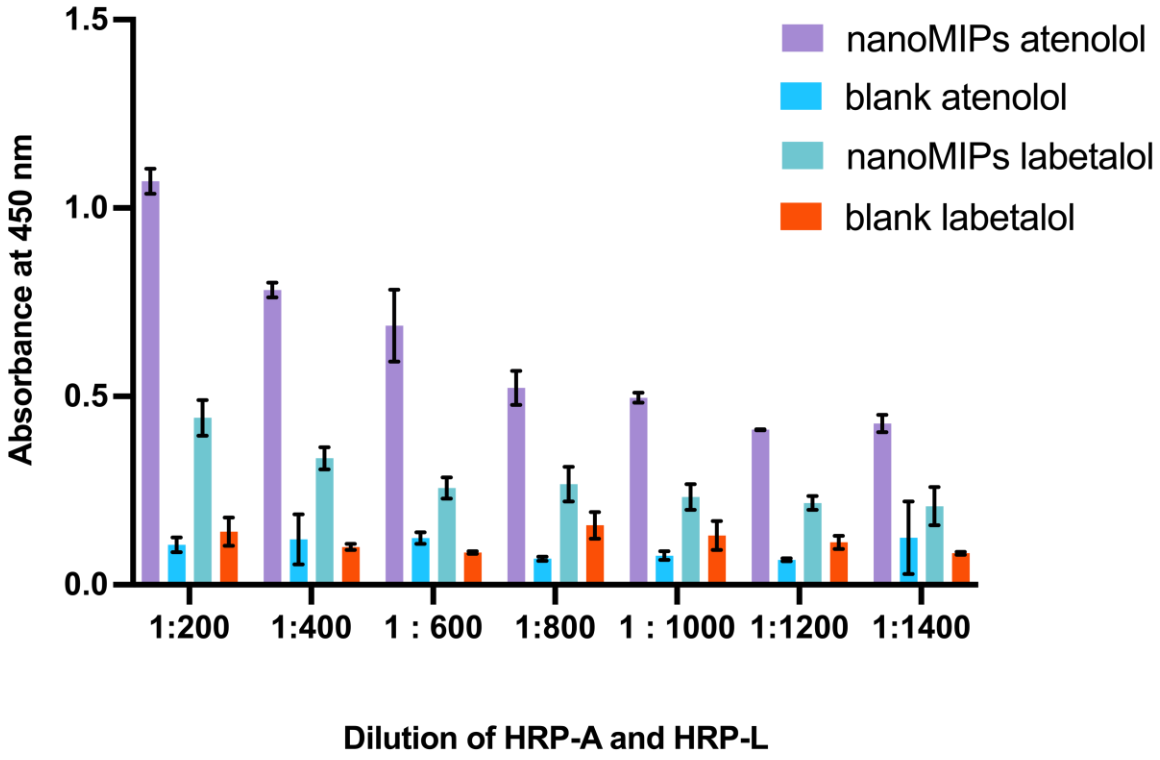

S6. The results obtained for different dilutions of the HRP-A, HRP-L conjugates and controls. Blue bars represent absorbance corresponding to the binding of the conjugate and nanoMIPs-A, green to the binding of the conjugate and nanoMIPs-L, red and purple are controls, without coated nanoMIPs.

Dilution HRP-Atenolol	Ratio	Dilution HRP-Labetalol	Ratio
1:200	10.10	1:200	3.14
1:400	6.49	1:400	3.34
1:600	5.55	1:600	3.00
1:800	7.57	1:800	1.69
1:1000	6.42	1:1000	1.78
1:1200	6.20	1:1200	1.93
1:1400	3.42	1:1400	0.25

S7. HRP-A conjugate and HRP-L conjugate dilutions in milli-Q water. Calculated ratios of the signal from wells with nanoMIPs to the controls (without coated nanoparticles) are presented on the right .

Ordinary one-way ANOVA multiple comparisons of Data 8 (A)

Number of families	1							
Number of comparisons per family	3							
Alpha	0,05							
Tukey's multiple comparisons test	Mean Diff.	95.00% CI of diff.	Significant?	Summary	Adjusted P Value			
atenolol solution vs. octopamine solution	0,7442	0.3266 to 1.162	Yes	***	0,0008	A-B		
atenolol solution vs. pseudofedrin solution	0,7214	0.3038 to 1.139	Yes	**	0,0010	A-C		
octopamine solution vs. pseudofedrin solution	-0,02282	-0.5050 to 0.4594	No	ns	0,9919	B-C		
Test details	Mean 1	Mean 2	Mean Diff.	SE of diff.	n1	n2	q	DF
atenolol solution vs. octopamine solution	1,059	0,3148	0,7442	0,1628	10	5	6,466	17
atenolol solution vs. pseudofedrin solution	1,059	0,3376	0,7214	0,1628	10	5	6,267	17
octopamine solution vs. pseudofedrin solution	0,3148	0,3376	-0,02282	0,1880	5	5	0,1717	17

S8 A). Analysis of variance ANOVA for data 8 (A)

Ordinary one-way ANOVA multiple comparisons of Data 8 (B)

Number of families	1							
Number of comparisons per family	3							
Alpha	0,05							
Tukey's multiple comparisons test	Mean Diff.	95.00% CI of diff.	Significant?	Summary	Adjusted P Value			
atenolol solution vs. octopamine solution	-0,04866	-0.1517 to 0.05442	No	ns	0,4432	A-B		
atenolol solution vs. pseudofedrin solution	-0,07148	-0.1746 to 0.03160	No	ns	0,1956	A-C		
octopamine solution vs. pseudofedrin solution	-0,02282	-0.1259 to 0.08026	No	ns	0,8277	B-C		
Test details	Mean 1	Mean 2	Mean Diff.	SE of diff.	n1	n2	q	DF
atenolol solution vs. octopamine solution	0,2661	0,3148	-0,04866	0,03864	5	5	1,781	12
atenolol solution vs. pseudofedrin solution	0,2661	0,3376	-0,07148	0,03864	5	5	2,616	12
octopamine solution vs. pseudofedrin solution	0,3148	0,3376	-0,02282	0,03864	5	5	0,8352	12

S8 B). Analysis of variance ANOVA for data 8 (B)

Ordinary one-way ANOVA multiple comparisons of Data 8 (C)

Number of families	1							
Number of comparisons per family	3							
Alpha	0,05							
Tukey's multiple comparisons test	Mean Diff.	95.00% CI of diff.	Significant?	Summary	Adjusted P Value			
labetalol solution vs. octopamine solution	0,1686	0.05583 to 0.2813	Yes	**	0,0061	A-B		
labetalol solution vs. pseudofedrin solution	0,1369	0.02411 to 0.2496	Yes	*	0,0198	A-C		
octopamine solution vs. pseudofedrin solution	-0,03173	-0.1445 to 0.08102	No	ns	0,7207	B-C		
Test details	Mean 1	Mean 2	Mean Diff.	SE of diff.	n1	n2	q	DF
labetalol solution vs. octopamine solution	0,4057	0,2371	0,1686	0,04038	4	4	5,904	9
labetalol solution vs. pseudofedrin solution	0,4057	0,2689	0,1369	0,04038	4	4	4,793	9
octopamine solution vs. pseudofedrin solution	0,2371	0,2689	-0,03173	0,04038	4	4	1,111	9

S8 C). Analysis of variance ANOVA for data 8 (C)

Ordinary one-way ANOVA multiple comparisons of Data 8 (D)

**Number of families**	**1**							
**Number of comparisons per family**	3							
**Alpha**	0,05							
**Tukey's multiple comparisons test**	Mean Diff.	95.00% CI of diff.	Significant?	Summary	Adjusted P Value			
**labetalol solution vs. octopamine solution**	0,01075	-0.03649 to 0.05799	No	ns	0,8049	A-B		
**labetalol solution vs. pseudofedrin solution**	0,01525	-0.03199 to 0.06249	No	ns	0,6530	A-C		
**octopamine solution vs. pseudofedrin solution**	0,004500	-0.04274 to 0.05174	No	ns	0,9619	B-C		
**Test details**	Mean 1	Mean 2	Mean Diff.	SE of diff.	n1	n2	q	DF
**labetalol solution vs. octopamine solution**	0,2329	0,2221	0,01075	0,01692	4	4	0,8985	9
**labetalol solution vs. pseudofedrin solution**	0,2329	0,2176	0,01525	0,01692	4	4	1,275	9
**octopamine solution vs. pseudofedrin solution**	0,2221	0,2176	0,004500	0,01692	4	4	0,3761	9

S8 D). Analysis of variance ANOVA for data 8 (D)
